# *Eudiplozoon nipponicum*: morphofunctional adaptations of diplozoid monogeneans for confronting their host

**DOI:** 10.1186/s40850-021-00087-5

**Published:** 2021-08-17

**Authors:** Andrea Valigurová, Naděžda Vaškovicová, Milan Gelnar, Magdaléna Kováčiková, Iveta Hodová

**Affiliations:** 1 Department of Botany and Zoology, Faculty of Science, Masaryk University, Kotlářská 2, 611 37 Brno, Czech Republic; 2 Institute of Scientific Instruments of the Czech Academy of Sciences, v.v.i., Královopolská 147, 612 64 Brno, Czech Republic

**Keywords:** Host-parasite interactions, Tegument, Musculature, Nervous system, Sensory structures, Excretory system, Secretion, Ultrastructure, Immunofluorescence, Freeze-etching

## Abstract

**Background:**

Monogeneans, in general, show a range of unique adaptations to a parasitic lifestyle, making this group enormously diverse. Due to their unique biological properties, diplozoid monogeneans represent an attractive model group for various investigations on diverse biological interactions. However, despite numerous studies, there are still gaps in our knowledge of diplozoid biology and morphofunctional adaptations.

**Results:**

In this study, we provide a comprehensive microscopic analysis of systems/structures involved in niche searching, sensing and self-protection against the host environment, and excretory/secretory processes in *Eudiplozoon nipponicum*. Freeze-etching enabled us to detect syncytium organisational features not visible by TEM alone, such as the presence of a membrane subjacent to the apical plasma membrane (separated by a dense protein layer) and a lack of basal plasma membrane. We located several types of secretory/excretory vesicles and bodies, including those attached to the superficial membranes of the tegument. Giant unicellular glands were seen accumulating predominantly in the apical forebody and hindbody haptor region. Muscle layer organisation differed from that generally described, with the outer circular and inner longitudinal muscles being basket-like interwoven by diagonal muscles with additional perpendicular muscles anchored to the tegument. Abundant muscles within the tegumentary ridges were detected, which presumably assist in fixing the parasite between the gill lamellae. Freeze-etching, alongside transmission electron and confocal microscopy with tubulin labelling, enabled visualisation of the protonephridia and nervous system, including the peripheral network and receptor innervation. Three types of receptor were identified: 1) uniciliated sensory endings with a subtle (or missing) tegumentary rim, 2) obviously raised uniciliated receptors with a prominent tegumentary rim (packed with massive innervation and muscles) and 3) non-ciliated papillae (restricted to the hindbody lateral region).

**Conclusions:**

This study points to specific morphofunctional adaptations that have evolved in diplozoid monogeneans to confront their fish host. We clearly demonstrate that the combination of different microscopic techniques is beneficial and can reveal hidden differences, even in much-studied model organisms such as *E. nipponicum*.

**Supplementary Information:**

The online version contains supplementary material available at 10.1186/s40850-021-00087-5.

## Background

There is no doubt that the study of adaptations to a parasitic lifestyle still represents a fascinating field of research. Monogeneans are among the most species-abundant groups of fish parasites [1] and, due to their unique life strategies and morphofunctional adaptations to ectoparasitism, they can be considered as highly successful. Due to their specific biological properties, monogenean parasites represent an attractive model group for investigations into biological interactions at organismal and cellular levels. Moreover, they represent a highly promising model for addressing a range of biological and ecological questions [2]. As monogeneans can cause serious health disorders, or even mortality, in fish maintained in both aquacultural and natural habitats [3], research on this parasitic group has wide potential for practical applications.

Oviparous monogenean, *Eudiplozoon nipponicum* (Goto, 1891) of the family Diplozoidae Palombi, 1949, represents a hematophagous ectoparasite of common carp (*Cyprinus carpio* L.). The family comprises obligatory gill parasites of freshwater fish with a unique reproductive strategy based on fusion of two diporpae followed by rearrangement and interconnection of their nervous systems. After somatic fusion, the two individuals grow and survive only in the form of an X-shaped single organism. Recent studies [4–6] have provided us with a good understanding of the diplozoid life cycle and their spectacular pairing strategy offers an excellent model for exploring various aspects of diplozoid monogenean biology [5–15].

Generally speaking, the tegument, or neodermis, typical of parasitic platyhelminths (Platyhelminthes, Neodermata), represents an important host-parasite interface involved in sensory, adhesive and immunological functions [16], and thus deserves more attention. The monogenean tegument exhibits specific morphofunctional and chemical adaptations to nutrient absorption and digestion, ion and water transport and protection, and has the potential to synthesise specific secretory bodies, enzymes and other substances essential for parasite growth, protection, self-maintenance and homeostasis. This site is densely interlaced with numerous nerve endings and gland ducts and bears various superficial structures dedicated to sensory perception and attachment to the host surface. Moreover, the tegument is associated with other organs, including the excretory system outlets [12], the innervation system [5, 9, 10, 15] and body wall musculature [6].

This study, directly linked to our previous microscopic studies on *E. nipponicum* [5, 6, 9–11], combines the approaches of confocal, electron and light microscopy, in order to provide a comprehensive study of body wall organisation and other structures deemed important for the formation of specific biological interactions with its host. This work represents the first published study on the application of freeze-etching on monogenean parasites.

## Results

### Body wall organisation and secretory/excretory structures

**Fig. 1:**
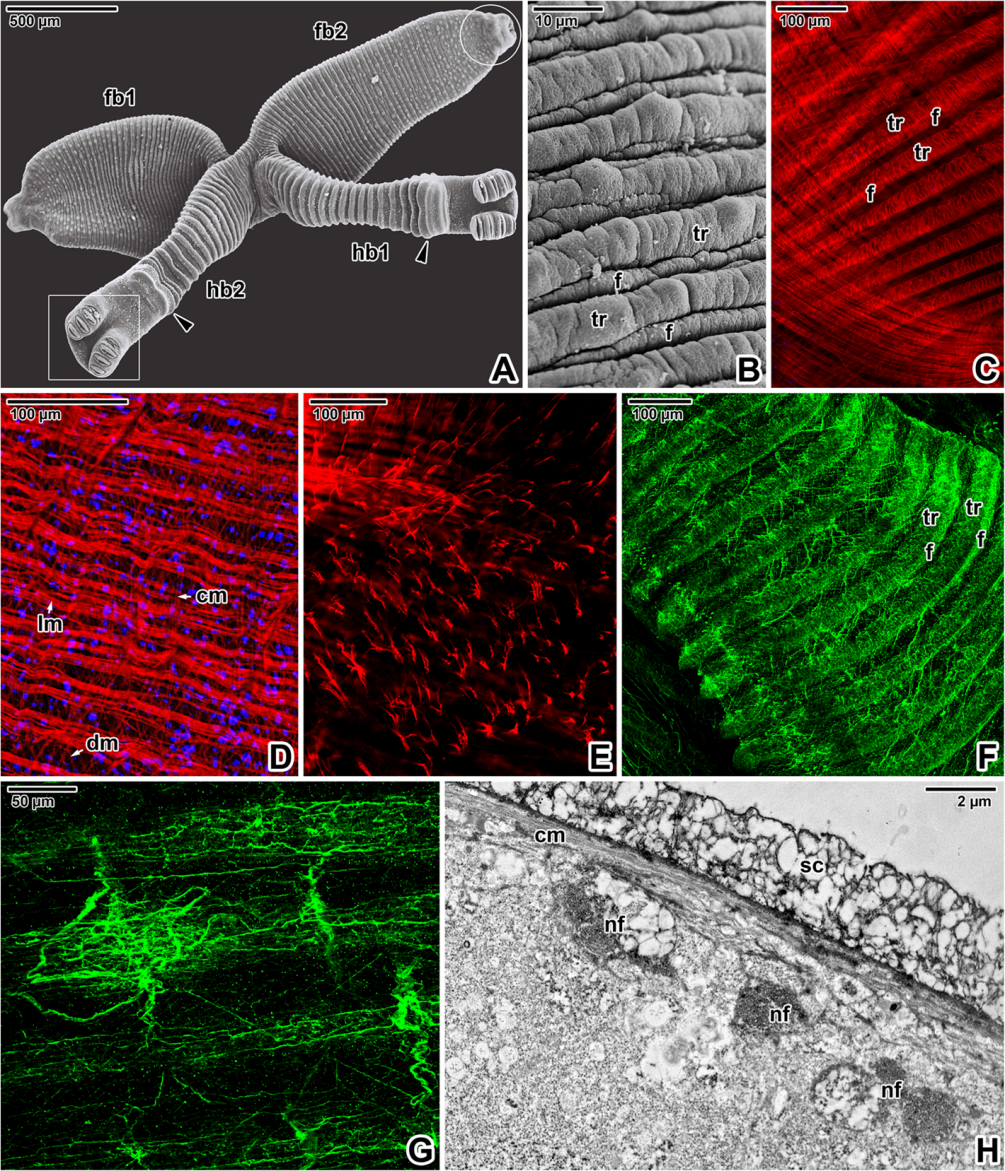
Body wall musculature and innervation in *Eudiplozoon nipponicum*. **A.** General view of an adult, with the hindbody bearing typical lobular extensions (arrowheads). The white circle indicates the area of the mouth and the white rectangle the area of the haptor. SEM. **B.** Detail of the forebody tegument surface, showing the tegumentary annular ridges and folds. SEM. **C.** Organisation of body wall musculature within the annular transverse ridges and folds. CLSM, phalloidin-TRITC. **D.** Detailed view of the body wall musculature, showing the circular, longitudinal and diagonal muscle fibres. CLSM, phalloidin-TRITC/Hoechst. **E.** Perpendicular muscle fibres. CLSM, phalloidin-TRITC. **F.** Peripheral nervous system subjacent to the tegument. CLSM, anti-α-tubulin-FITC. **G.** High magnification of the tegument innervation. CLSM, anti-α-tubulin-FITC. **H.** Nerve nodes located deeper within the tegument. TEM. *cm* – circular muscle fibres, *dm* – diagonal muscle fibres, *f* – folds, *fb1*– forebody 1*, fb2* – forebody, *hb1* – hindbody 1, *hb2* – hindbody 2, *lm* – longitudinal muscle fibres, *nf* – peripheral nerve fibres subjacent to the circular musculature, *sc* – syncytium, *tr* – annular transverse ridges

**Fig. 2:**
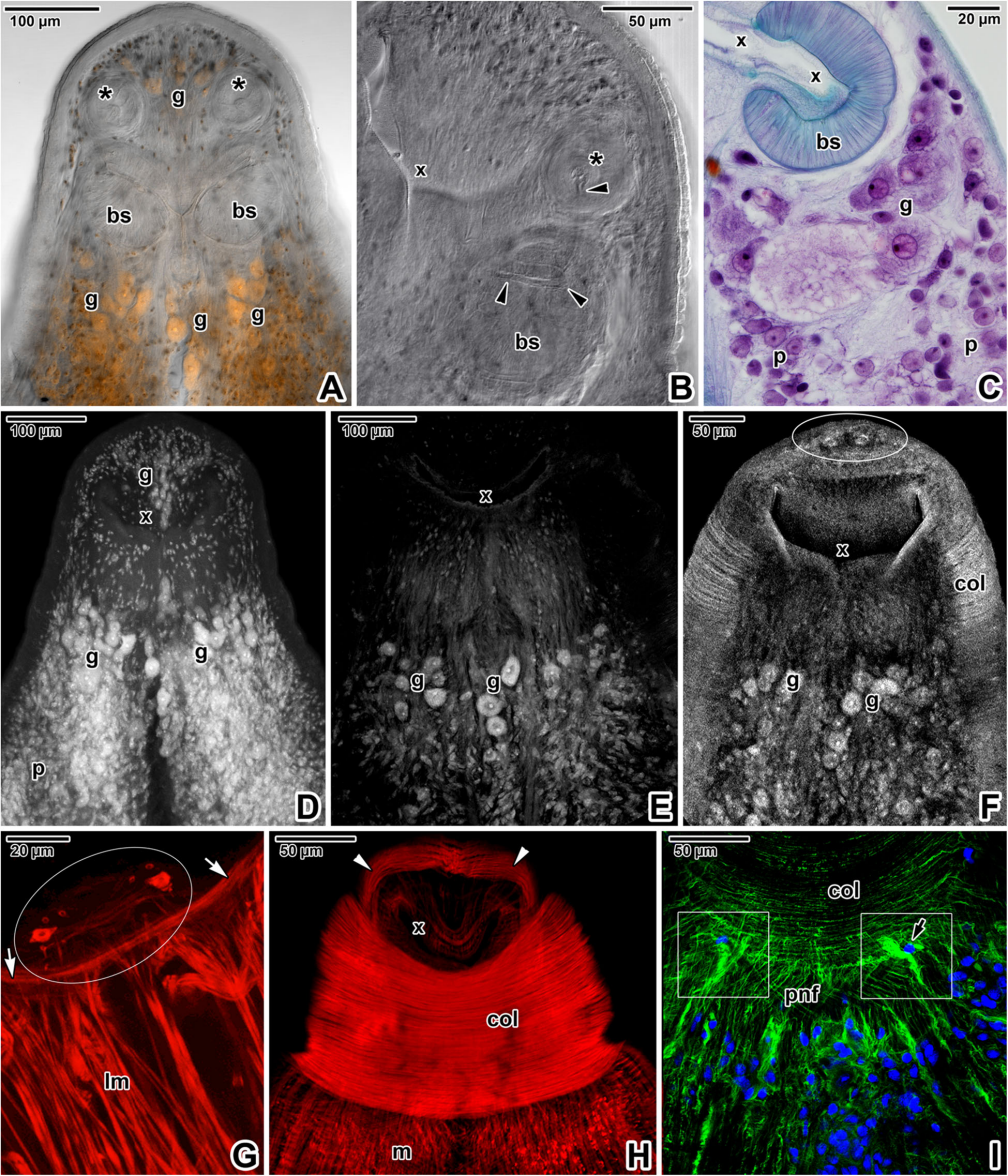
Organisation of the apical forebody region in *Eudiplozoon nipponicum*, with emphasis on glandular structures. **A.** General view of the forebody in the area of the mouth cavity, showing localisation of paired buccal suckers and GMO. Note the gland cell accumulations around this structure. CLSM, hydrochloric carmine staining in combination with transmission light. **B.** Detail of mouth opening with prominent buccal sucker and GMO connected to a canal. CLSM, transmission light. **C.** Histological section showing a lateral view of the forebody, with the muscular buccal sucker and several giant gland cells located below it. LM, MT. **D-F.** Forebody stained with hydrochloric carmine showing distribution of putative unicellular glands. Note the apically located circular structures in 2F. CLSM, output image not coloured. **G.** The apical part of the forebody with two symmetric groups of circular structures (encircled). CLSM, phalloidin-TRITC. **H.** Micrograph showing the mouth opening and a retracted collar with well-developed circular muscle fibres. CLSM, phalloidin-TRITC. **I.** Innervation of the collar and subjacent tegument. Note the paired nerve nodes (white rectangles) located at the base of the collar. CLSM, anti-α-tubulin-FITC. *arrow* – flame cell, *arrowheads* –internal canal attached to the GMO, *asterisk* – GMO, *bs* – buccal sucker, *col* – forebody collar, *g* – gland cells, *lm* – bundles of longitudinal muscle fibres, *m* – body wall musculature, *p* – parenchyma, *pnf* – peripheral nerve fibres subjacent to the tegument, *white arrowheads* – apical band of musculature, *white arrows* – muscle fibres arranged circularly around the border of the mouth opening, *x* – mouth opening

The X-shaped adults of *E. nipponicum* have a subterminal mouth and a fully developed haptor with two rows of the fourth clamps (Fig. 1A). In adults, the hindbody bears prominent lobular extensions (Fig. 1A). Compared to the diporpae or juvenile stages (with ridges and folds gradually becoming more distinct during development) [see Additional file 1], the forebody is covered with well-developed, annular transverse ridges alternating with lower folds, which are superimposed onto the ridges (Fig. 1B). The ridges are discontinuous at the lateral margins. The ridges and folds of the fused adult are continuous at the junction area. Phalloidin labelling of filamentous actin (F-actin) showed typical organisation of the body wall musculature, consisting of outer circular and inner longitudinal muscle fibres, while the diagonal musculature comprised two components oriented perpendicular to one another that pass through the circular and longitudinal muscles (Fig. 1C-D). Additional perpendicular muscle fibres anchor to the tegument (Fig. 1E). Consistent with the musculature organisation (Fig. 1C), fluorescent labelling of the nervous system using the α-tubulin antibody revealed a dense mesh of peripheral nerve fibres innervating the tegumentary ridges and folds (Fig. 1F-G) and two deeper located ventral nerve cords interconnected by the transverse commissures (Fig. 9B). Accordingly, bundles of unmyelinated nerve fibres with vesicles (some with electron-dense contents) were observed in ultrathin sections located beneath the circular musculature (Fig. 1H). Hydrochloric carmine staining for confocal laser scanning microscopy (CLSM; Fig. 2A, D-F) combined with histology (Fig. 2C) revealed the presence of numerous giant cells with prominent nuclei, considered to be unicellular glands. These were accumulated below buccal suckers and around the pharynx as well as between the glandulo-muscular organs (GMO) located anterior to the buccal suckers. A long canal arises from the GMO and run to the buccal sucker located in the mouth cavity (Fig. 2B). The ventral part of the apical forebody is equipped with symmetrically organised areas with several circular structures (a central large structure surrounded by several smaller structures; Fig. 2F-G). The apical area of the forebody has a pronounced mouth lined with a thick collar of circular musculature (Fig. 2F, H) and is densely innervated with several areas of fluorescence hyperintensity resembling nerve nodes (Fig. 2I).

**Fig. 3:**
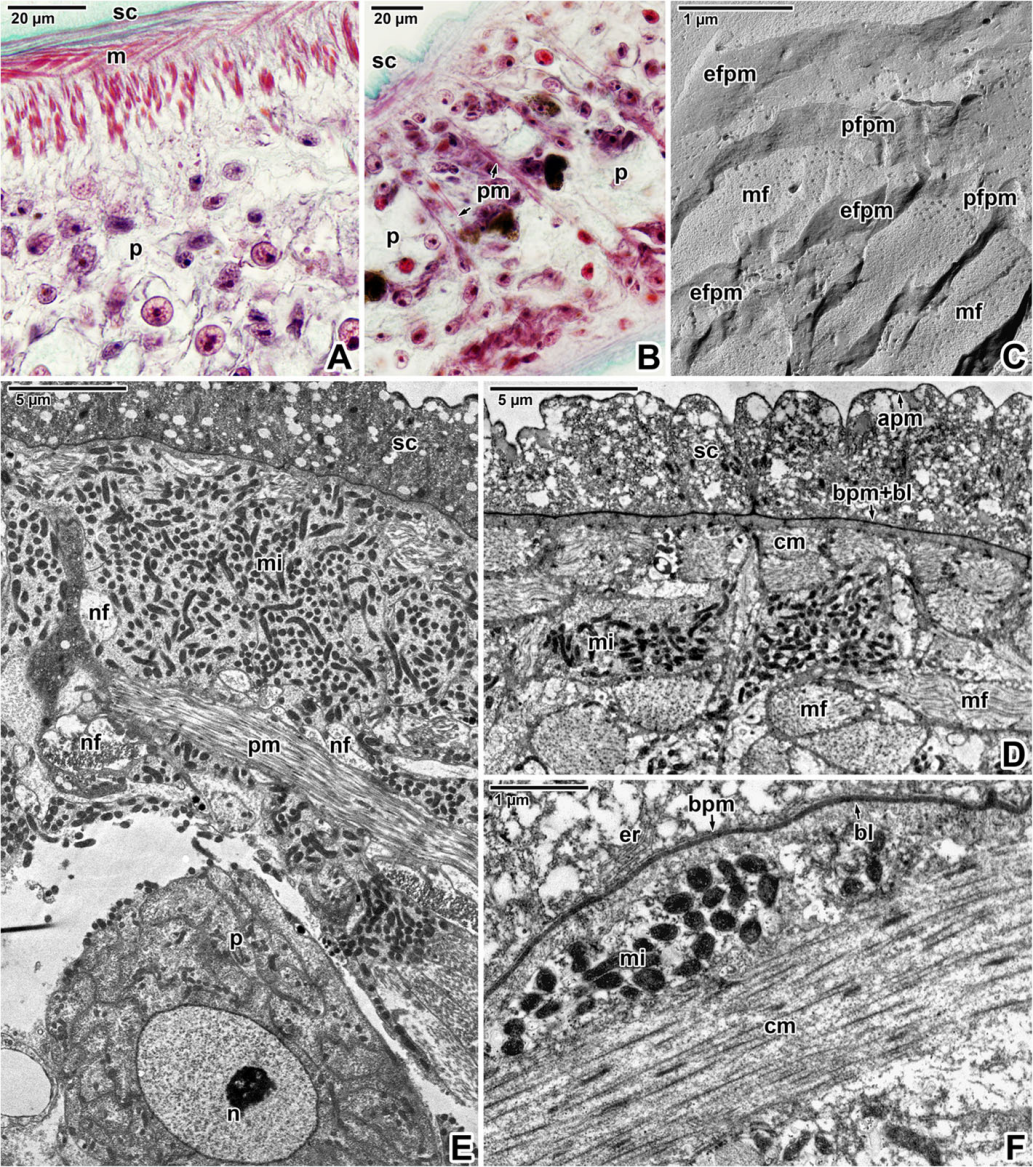
Forebody body wall organisation in *Eudiplozoon nipponicum*. **A-B.** Histological sections showing the forebody wall musculature and parenchyma. LM, MT. **C.** Detail of freeze-fractured muscle bundles. FE, TEM. **D.** Syncytium and body wall musculature. TEM. **E.** General view of tegument organisation. TEM. **F.** High magnification of the basal lamina underlain by a circular muscle layer. Note the endoplasmic reticulum in the syncytium, just above the basal plasma membrane. TEM. *apm* – apical plasma membrane, *bl* – basal lamina, *bpm* – basal plasma membrane, *cm* – circular muscle fibres, e*fpm –* exoplasmic fracture face of the plasma membrane, *er* – endoplasmic reticulum, *m* – body wall musculature, *mf* – bundles of myofibrils, *mi* – mitochondria, *n* – nucleus, *nf* – nerve fibres, *p* – parenchyma, *pfpm* – protoplasmic fracture face of the plasma membrane, *pm* – perpendicular muscle fibres, *sc* – syncytium

**Fig. 4:**
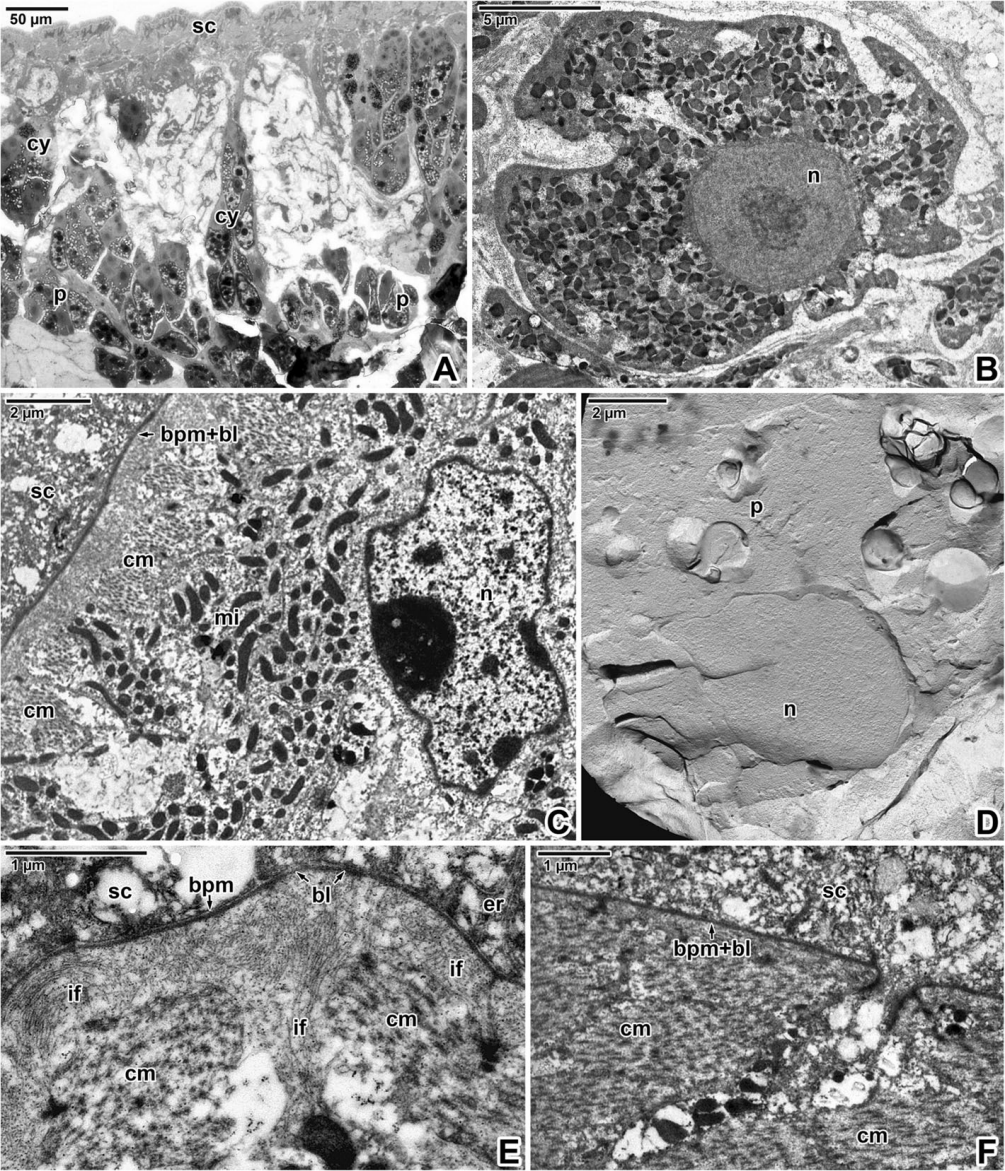
Forebody tegument organisation in *Eudiplozoon nipponicum*. **A.** General view of parenchyma, showing cytons connected to the syncytium by thin cytoplasmic extensions. TEM. **B.** A cyton with secretory bodies. TEM. **C.** Detailed view of musculature containing numerous mitochondria. TEM. **D.** A freeze-fractured parenchyma cell with a large nucleus. FE, TEM. **E.** Interstitial fibres adjacent to the basal lamina. TEM. **F.** High magnification showing the interruption of basal lamina. TEM. *bl* – basal lamina, *bpm* – basal plasma membrane, *cm* – circular muscle fibres, *cy* – cyton, *er* – projections of endoplasmic reticulum, *if* – interstitial fibres, *mi* – mitochondria, *n* – nucleus, *p* – parenchyma, *sc* – syncytium

**Fig. 5:**
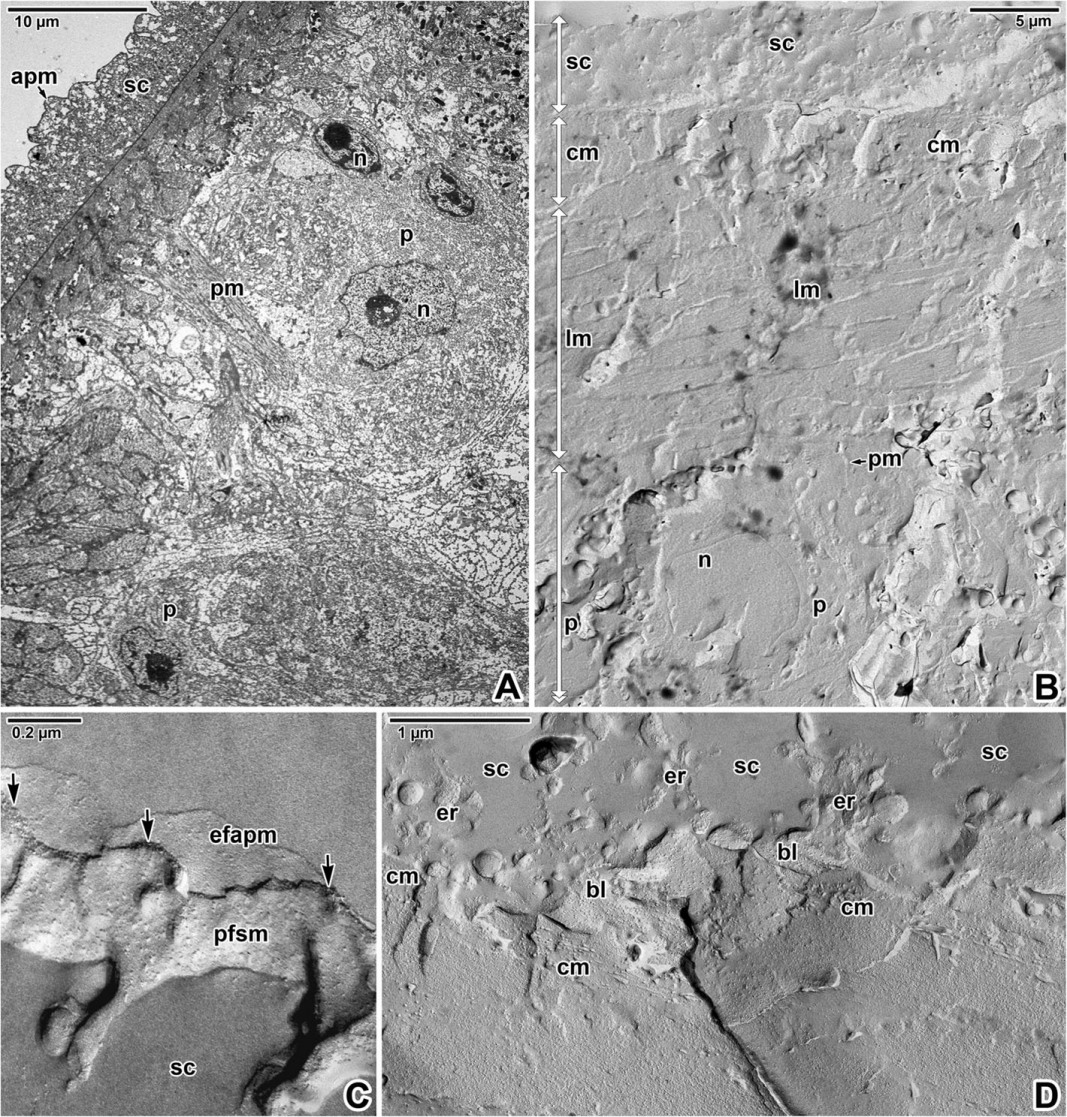
Forebody tegument organisation in *Eudiplozoon nipponicum*. **A.** General view of tegument organisation. TEM. **B.** General view of freeze-fractured tegument. The white double-sided arrows demark the thickness of individual layers comprising the syncytium, musculature and part of the parenchyma. FE, TEM. **C.** A detail of the fractured apical plasma membrane and subjacent membrane. FE, TEM. **D.** Fractured tegument, showing the basal lamina separating the syncytium from the body wall musculature. FE, TEM. *apm* – apical plasma membrane, *bl* – basal lamina, *black arrows* – dense protein layer, *cm* – circular muscle fibres, *efapm* – exoplasmic fracture face of the apical plasma membrane, *er* – projections of endoplasmic reticulum, *lm* – longitudinal muscle fibres, *n* – nucleus, *p* – parenchyma, *pfsm* – protoplasmic fracture face of the subjacent membrane, *pm* – perpendicular muscle fibres, *sc* – syncytium

**Fig. 6:**
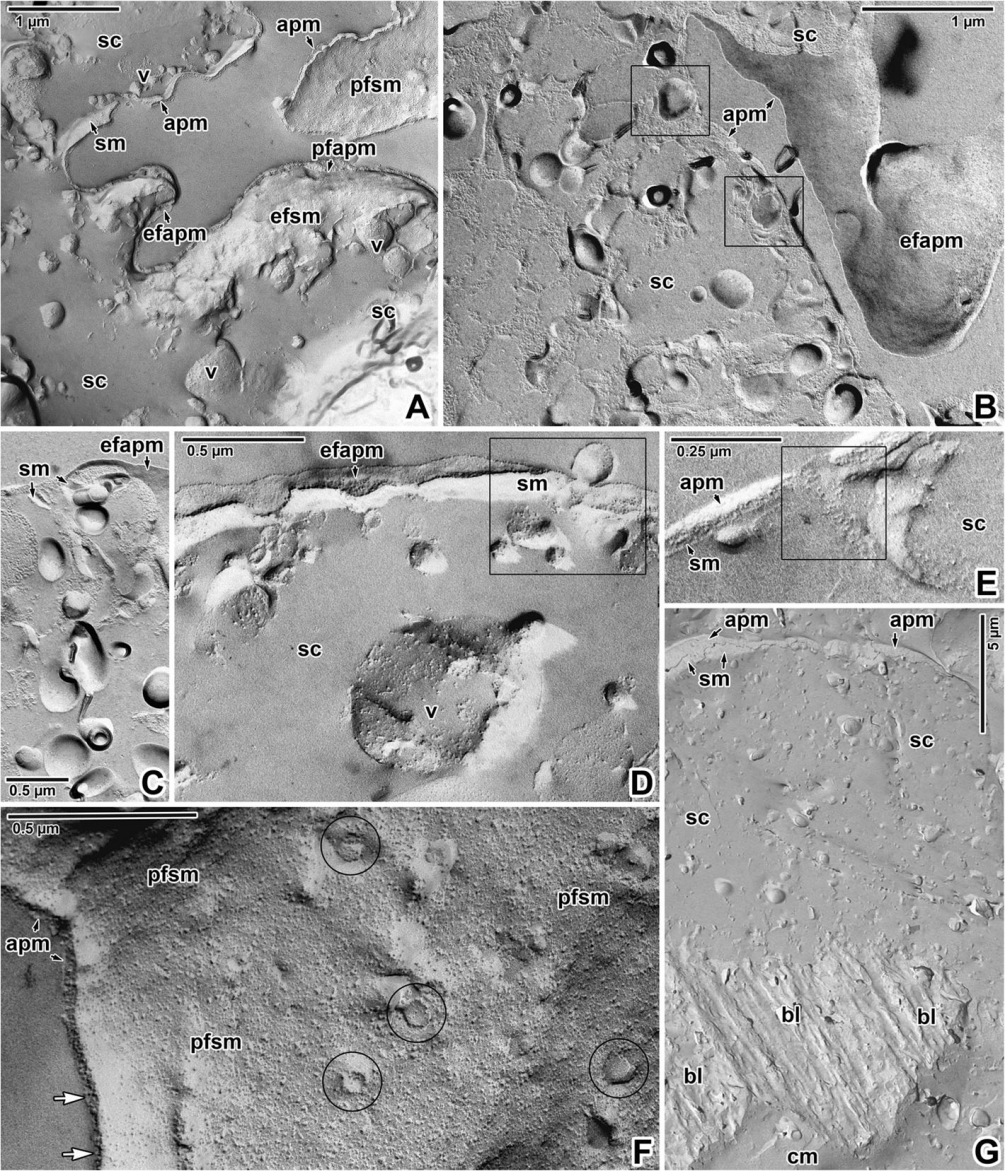
Forebody tegument organisation in *Eudiplozoon nipponicum*. **A.** The outer zone of the syncytium lined by the apical plasma membrane and subjacent membrane. FE, TEM. **B.** A view of the syncytium, with putative secretory vesicles (black rectangles) connecting the subjacent and apical plasma membranes. FE, TEM. **C.** The putative secretory vesicles in detail. FE, TEM. **D-E.** Freeze-fractured apical plasma and subjacent membranes interrupted by secretory vesicles (black rectangles). FE, TEM. **F.** Protoplasmic fracture face of the subjacent membrane, revealing the presence of vesicle attachment sites (black circles). Note the dense layer of proteins (white arrows) separating the apical plasma membrane and subjacent membrane. FE, TEM. **G.** General view of the syncytium and the basal lamina. FE, TEM. *apm* – apical plasma membrane, *bl* – basal lamina, *cm* – circular muscle fibres, *efapm* – exoplasmic fracture face of the apical plasma membrane, *efsm* – exoplasmic fracture face of the subjacent membrane, *pfapm* – protoplasmic fracture face of the apical plasma membrane, *pfsm –* protoplasmic fracture face of the subjacent membrane, *sc* – syncytium, *sm –* subjacent membrane, *v* – vesicles, *white arrow* – dense protein layer

The tegument showed typical organisation with distinctly separated layers of a foam-like syncytium containing various types of vesicles and inclusions, body wall musculature and a deeper located parenchyma (Fig. 3A-F). The syncytium, with abundant scattered secretory bodies, is delimited by an apical plasma membrane and basal plasma membrane underlain by fibrous basal lamina (Fig. 3D-F). Histological and transmission electron microscopic analyses of the body wall musculature confirmed the presence of an outer circular, inner longitudinal and several diagonal muscle fibres arranged in bands. In addition, long and thin perpendicular muscles run through the parenchyma (Fig. 3B, E). The muscles contained abundant mitochondria (Figs. 3D-F, 4C) with well-developed cristae. Nerve fibrils were detected adjacent to the muscles in some ultrathin sections (Fig. 3E). During the development of diporpae and juveniles into the adult stage, the parenchymal cells become more abundant, though the connective tissue remain sparse [Additional file 1]. The parenchymal cells in the adult stage are characterised by dense cytoplasm and a prominent nucleus with a single, relatively small nucleolus (Figs. 3A, E, 4D). The surrounding connective tissue consists of a matrix and interstitial fibres. The syncytium is connected to the cell bodies (cytons) (Fig. 4A-B) lying beneath the body wall musculature. These are rich in secretory bodies and have cytoplasmic extensions that are interwoven between the muscle fibres. Accordingly, interruptions in the basal lamina can be found in ultrathin sections (Fig. 4F). Furthermore, a continuous layer of interstitial material with randomly oriented fibres is located just beneath the basal lamina and around the subjacent muscle fibres anchored to the tegument (Fig. 4E). Freeze-etching of the tegument confirmed the organisation observed in ultrathin sections (Fig. 5A-B) and revealed the presence of two closely associated membranes covering the outer zone of the syncytium, i.e. apical plasma membrane separated by a dense layer of proteins from a subjacent membrane (Figs. 5C, 6A-F). Groups of vesicles or possible secretory bodies accumulate in the syncytium just beneath these two membranes (Fig. 6B-C), while others interrupt them (Fig. 6D-E). The protoplasmic face of the subjacent membrane shows circular, pore-like structures that most likely represent the sites where membrane-bound vesicles attach to the superficial membrane of the parasite’s tegument (Fig. 6F). While ultrathin sections revealed the putative basal plasma membrane as located between the innermost site of the syncytium and the basal lamina (Fig. 4E), no similar structure was obvious in fractured replicas (Fig. 6G). Instead, abundant separating vesicles, most likely of endoplasmic reticulum origin, cover the outer surface of the basal lamina (Fig. 5C-D).

**Fig. 7:**
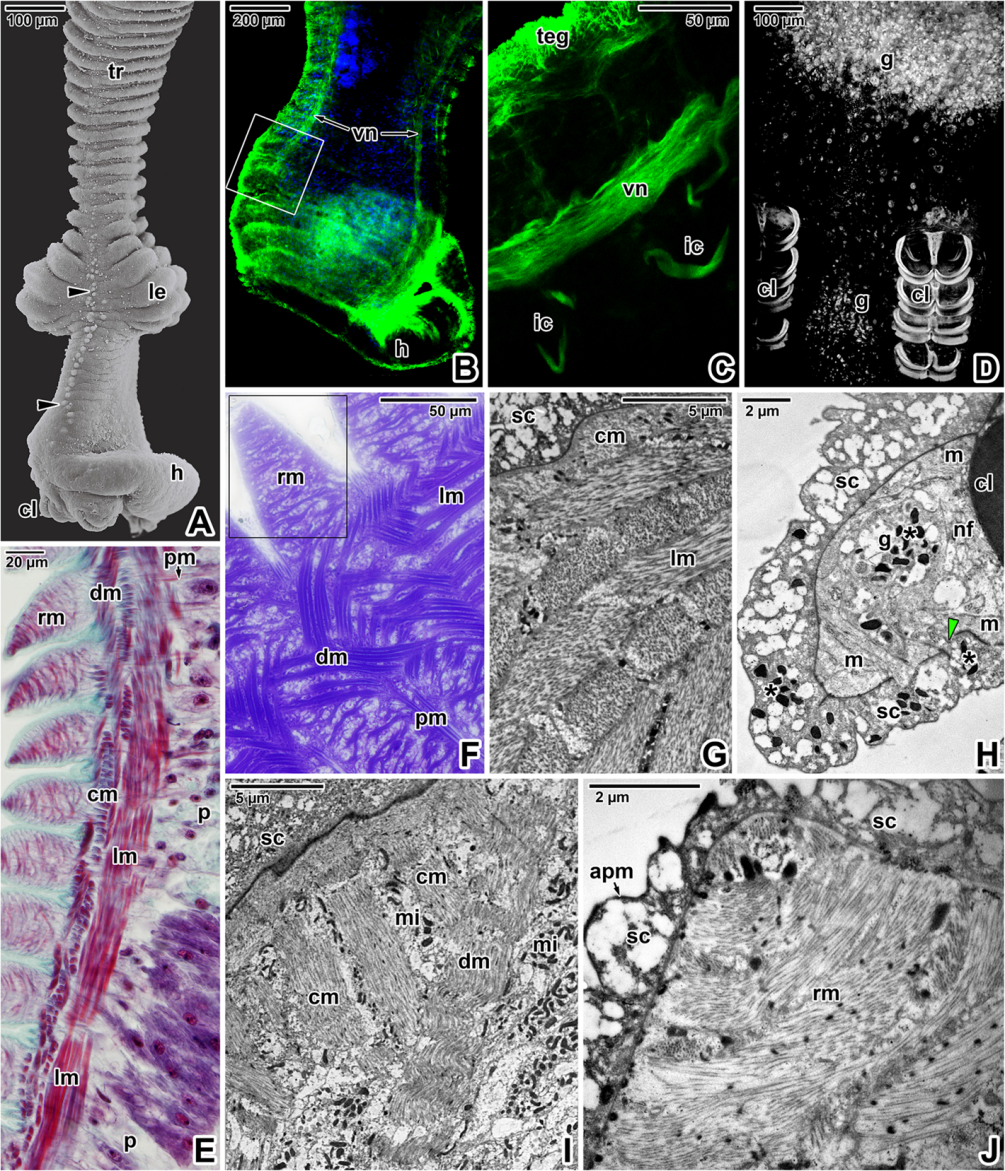
Hindbody tegument organisation and innervation in *Eudiplozoon nipponicum*. **A.** General view of the hindbody bearing the haptor with clamps. Note the tegumentary annular ridges, lobular extensions and the row of non-ciliated papillae. SEM. **B.** A general view of the hindbody nervous system. CLSM, anti-α-tubulin-FITC/Hoechst. **C.** Detail of hindbody nerves, comprising the ventral cord and innervation to the haptor clamps. The micrograph shows the area marked by a white rectangle in B. CLSM, anti-α-tubulin-FITC. **D.** Hindbody stained with hydrochloric carmine, showing putative unicellular glands. CLSM, output image not coloured. **E.** Histological section showing the hindbody transverse tegumentary annular ridges and body wall musculature. LM, MT. **F.** Semithin section of tegument with ridges (black rectangle), revealing the organisation of individual muscle layers. LM, Toluidine blue. **G.** Ultrathin section showing circular and longitudinal muscles. TEM. **H.** Ultrastructural organisation of the haptor tegument. Note the gland cell with numerous secretory bodies and released secretory bodies within the syncytium. TEM. **I.** Fine structure of the hindbody tegument and body wall musculature. TEM. **J.** Detailed view showing the fine structure of the tegumentary ridge (similar to the area demarked by a black rectangle in F). TEM. *apm* – apical plasma membrane, *asterisk* – secretory bodies, *black arrowheads –* non-ciliated papillae, *cl* – clamp, *cm* – circular muscle fibres, *dm* – diagonal muscle fibres, *g* – gland cells, *green arrowhead* – interruption of basal lamina, *h* – haptor, *ic –* innervation of the haptor clamps, *le* – lobular extensions, *lm* – longitudinal muscle fibres, *m* – body wall musculature, *mi* – mitochondria, *nf* – nerve fibres innervating the clamp (nerve plexus), *p* – parenchyma, *pm* – perpendicular muscle fibres, *rm* – muscle fibres within tegumentary ridges, *sc* – syncytium, *teg* – tegument, *tr* – annular transverse ridges, *vn* – ventral nerve cord

Compared to the forebody (Fig. 1A-B), the tegumentary annular transverse ridges covering the parasite’s hindbody are more developed, making the alternating lower folds almost invisible in this area (Fig. 7A). In addition, the hindbody tegument surface bears a row of papilla-like non-ciliated structures running longitudinally along each of the hindbody lateral sides, from the anterior part of the lobular extensions to the haptor (Fig. 7A). The two prominent ventral nerve cords reach the haptor region and are connected to the clamp’s innervation (Fig. 7B-C, and also seen in TEM - Fig. 7H). As in the forebody, the tegument of the hindbody shows dense peripheral innervation (Fig. 7B-C). Hydrochloric carmine staining revealed the presence of gland-like cells in the haptor region, these being considerably smaller than those in the forebody (Fig. 7D). The tegument of the haptor shows the same organisation as in the other body parts (Fig. 7E-I), though further enriched by the presence of muscles detected in the tegumentary annular ridges (Figs. 7E-F, J). The tegument covering the haptor has reduced parenchyma. Large dense secretory bodies were seen in the gland-like cell within the parenchyma, while other (most likely secreted) bodies were located free in the syncytium (Fig. 7H).

**Fig. 8:**
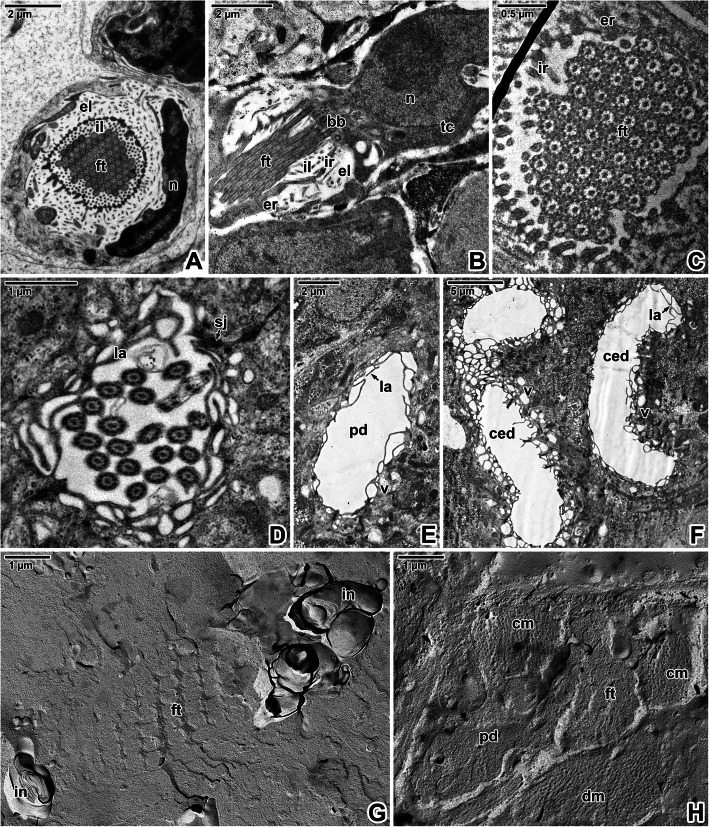
Excretory system in *Eudiplozoon nipponicum*. **A.** Cross-sectioned flame bulb at the level of the basal part of the cilia. Note the terminal cell with a large nucleus and the flame bulb with a flame tuft flagellation. TEM. **B.** Longitudinally sectioned flame tuft flagellation showing the non-striated rootlets, external ribs arising from the proximal canal cell, internal leptotriches and internal ribs arising from the terminal cell. TEM. **C.** Cross-sectioned flame cell in the area of the weir apparatus. TEM. **D.** Region of the protonephridial duct epithelium, showing the lateral flame. TEM. **E.** Cross-sectioned protonephridial duct. TEM. **F.** A section of the convoluted excretory duct. TEM. **G.** Fractured flame cell with neighbouring innervation. FE, TEM. **H.** Fractured flame cell and protonephridial duct with neighbouring muscle fibres. FE, TEM. *bb* – basal bodies, *ced* – convoluted excretory ducts, *cm* – circular muscle fibres, *dm* – diagonal muscle fibres, *el* – external leptotriches, *er* – external ribs, *ft* – flame tuft, *il* – internal leptotriches, in – innervation, *ir* – internal ribs, *la* – lamellae, *n* – nucleus of terminal cell, *pd* – protonephridial duct, *sj* – septate junction, *tc* – terminal cell, *v* – vacuolated epithelium

Ultrathin sections and freeze-etching confirmed that the terminal part of the protonephridia, embedded within layers of body wall musculature and parenchyma, comprises a flame cell composed of a terminal cell with a prominent nucleus and a flame bulb with ciliary tuft (Fig. 8A-C, G-H). The flame bulb consists of a terminal cell, proximal canal cell and cytoplasmic outgrowths (internal and external ribs) which are interdigitated and form the filtration apparatus (weir). Numerous internal leptotriches arising in the terminal cell extend into the lumen of the flame bulb, while just a few external leptotriches arise from the proximal canal cell. The tuft of cilia arising from the terminal cell is anchored by short non-striated rootlets. Protonephridial ducts (Fig. 8D-E) and convoluted excretory ducts (Fig. 8F) were lined with a strongly vacuolated epithelium enlarged by long and thin luminal projections (lamellae). Using the α-tubulin antibody, we visualised tufts of flame cells with a closely located prominent nucleus in the terminal cell (Fig. 10I-J).

### Neurosensory structures

**Fig. 9:**
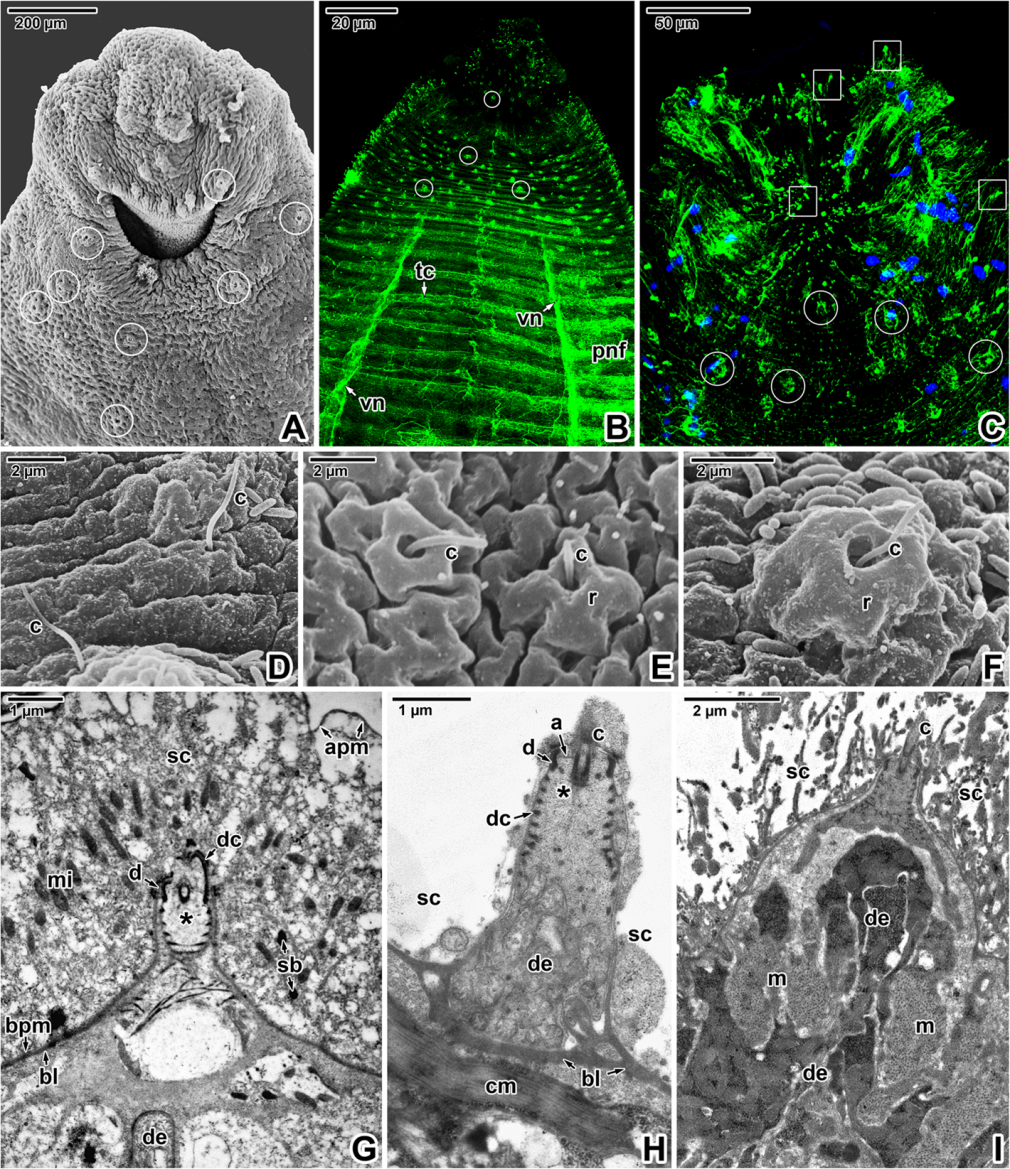
Sensory structures in *Eudiplozoon nipponicum*. A. General view of the forebody with its prominent mouth. White circles indicate some of the uniciliated sensory structures. SEM. **B.** General view of the forebody, showing the nervous system and distribution of sensory structures. White circles indicate innervation of some of the uniciliated sensory structures. CLSM, anti-α-tubulin-FITC. **C.** A more superficial optical section showing the distribution of sensory structures. White circles indicate examples of their innervation and white rectangles demark sensory cilia. CLSM, anti-α-tubulin-FITC/Hoechst. **D-F.** Uniciliated sensory structures in the forebody – without a tegumentary rim (D), with a subtle rim (E) and with a prominent rim (F). SEM. **G-H.** Uniciliated sensory structures interrupting the forebody (G) and hindbody tegument (H). TEM. **I.** Uniciliated sensory structure with a prominent tegumentary rim interrupting the forebody tegument. TEM. *a* – cilium anchoring, *apm* – apical plasma membrane, *bl* – basal lamina, *black asterisk* – basal body of the cilium, *bpm* – basal plasma membrane, *c* – cilium, *cm* – circular muscle fibres, *d* – desmosome, *dc* – dense collar, *de* – dendrite, *m –* muscles, *mi* – mitochondria, *pnf* – peripheral nerve fibres, *r* – raised rim, *sb* – secretory bodies, *sc* – syncytium, *tc* – transverse commissure, *vn* – ventral nerve cord

**Fig. 10:**
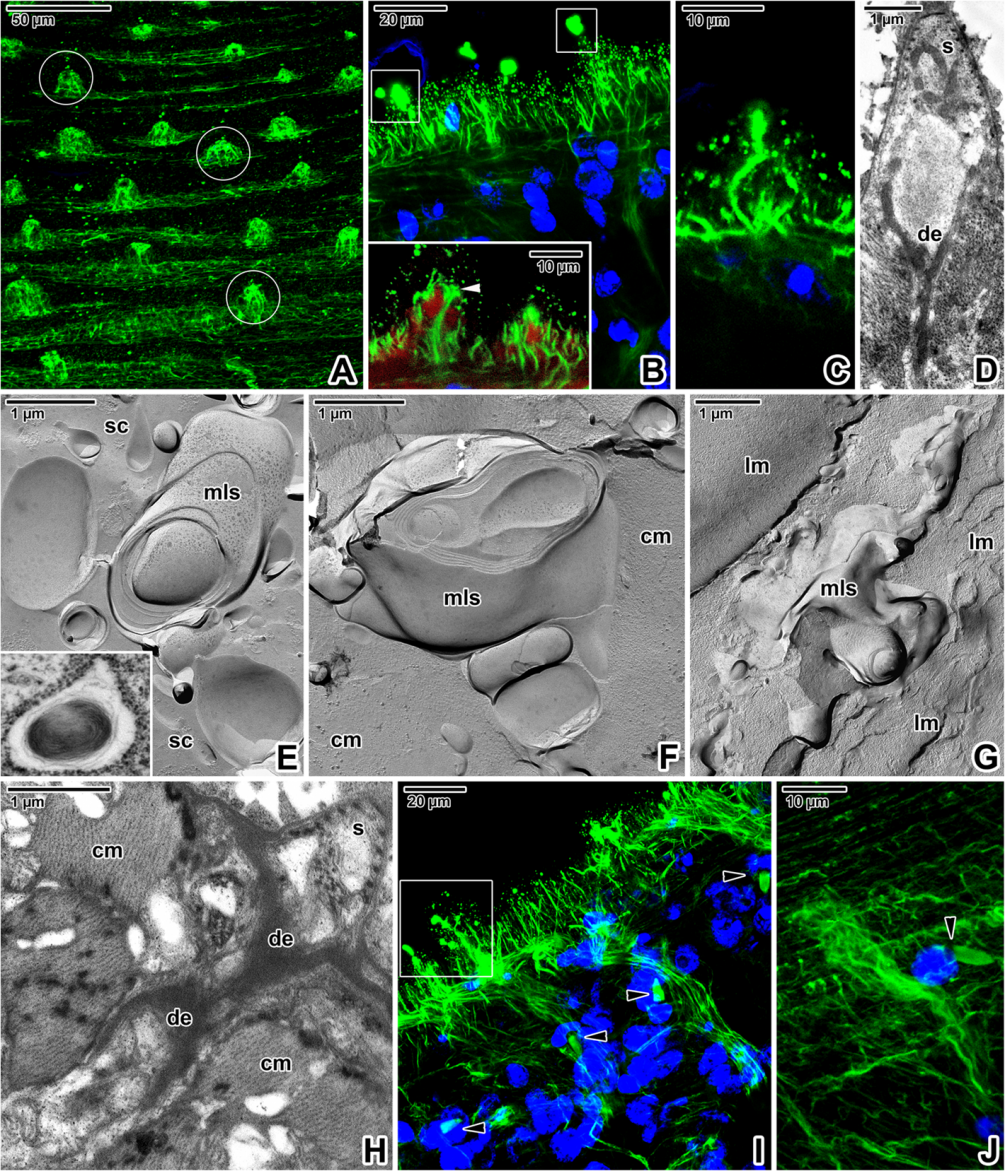
Neurosensory and excretory structures in *Eudiplozoon nipponicum*. **A.** General view of the forebody tegument, showing the distribution of sensory structures. White circles indicate a few examples of their innervation. CLSM, anti-α-tubulin-FITC. **B.** Detailed view of the tegument with labelled sensory structures. White rectangles indicate sensory cilia. CLSM, anti-α-tubulin-FITC/Hoechst. The inset in B shows two uniciliated receptors in another optical section, with shown phalloidin-TRITC counterstaining. Note the raised receptor with prominent innervation surrounded by muscles on the left of the image. **C.** High magnification showing uniciliated sensory structure with innervation. CLSM, anti-α-tubulin-FITC/Hoechst. **D.** A uniciliated sensory structure with visible dendrite. TEM. **E-G.** Putative innervation of sensory structures detected in the syncytium above the basal lamina (E) and between the circular (F) and longitudinal (G) muscle fibres. FE, TEM. The inset in E shows a putative nerve fibre in cross-ultrathin section. TEM. **H.** A longitudinal section of the innervation of a uniciliated sensory structure. TEM. **I.** Detailed view of the tegument, showing the sensory structures with innervation (white rectangle) and flame cells (arrowheads). CLSM, anti-α-tubulin-FITC/Hoechst. **J.** Detail of nerve fibres and a single flame cell. CLSM, anti-α-tubulin-FITC/Hoechst. *black arrowheads* – flame cells, *cm* – circular muscle fibres, *de* – dendrite, *lm* – longitudinal muscle fibres, *mls* – structures resembling nerve fibres with myelin sheaths, *s* – sensory structure, *sc* – syncytium, *white arrowhead* – centrally located innervation

Abundant sensory structures, with a single long cilium interrupting the syncytium, are accumulated predominately in the forebody region and especially around the mouth (Fig. 9A-C). While some of the uniciliated receptors possess a massive raised circular rim (Fig. 9F, I), the tegumentary rim of the others is subtle (Fig. 9E, G-H) or completely absent (Fig. 9D). Uniciliated receptors with no obvious raised rim are abundant on the entire forebody. The bases of uniciliated receptors with prominent raised rims are packed with massive innervation and muscles (Fig. 9I). The nerve bulbs of all observed uniciliated receptors anchored within the syncytium by a desmosome are characterised by the presence of a spirally arranged dense collar (Fig. 9G, H). The peripheral nerve fibres are associated with sensory structures bearing a tubulin-rich cilium (Figs. 9B-C, 10A-C, I). A centrally located innervation surrounded by muscles is visible in the centre of more raised uniciliated receptors (inset in Fig. 10B), with their morphology corresponding to uniciliated receptors with a prominent tegumentary rim (Fig. 9I). Innervation of the sensory structures is also visible in ultrathin sections (Figs. 9G-I, 10D, H). Similarly, freeze-etching revealed the presence of structures conspicuously resembling the myelin sheaths wrapped around the nerve axon (Fig. 10E-G). These putative nerve fibres are found in the syncytium close to the basal lamina or deeper within the body wall musculature, and thus may represent innervation of the sensory structures. Corresponding structures are occasionally seen in ultrathin sections (inset in Fig. 10E).

## Discussion

Ectoparasitic diplozoid monogeneans exhibit considerable functional and morphological adaptations to their parasitic lifestyle. This study focuses on body wall organisation and related structures involved in niche searching, host confrontation and self-protection against the environment and excretory/secretory processes.

### Self-protection against the environment and excretory/secretory processes

The Neodermata have evolved a unique body covering (tegument) on their surface with numerous functions, including provision of external body support, contributing to the transfer of nutrients and their conversion to energy and protection against host immune responses, enzymes, secretion, excretion and osmoregulation, as well as providing a sensory function [17]. The tegument is the primary interface essential for host-parasite interaction and, as such, exhibits unique modifications related to both nutrition and the worm’s parasitic strategy. Generally speaking, the tegument and related structures of parasitic flatworms exhibit significant variability in organisation and basic function. Unlike cestodes, digeneans and some other monogeneans [17], where the external surface of the tegument is covered with highly specialised microvilli (microtriches) projecting from the apical plasma membrane that are essential for nutrient absorption and elimination of waste materials, the surface of the apical plasma membrane in *E. nipponicum* is smooth. However, the tegument of *E. nipponicum*, *Paradiplozoon homoion* (Bychowsky & Nagibina, 1959) and a few other species is equipped with shallow pits [11, 15, 18]. The prominent annular ridges and extensions of the *E. nipponicum* hindbody are considered to be important for attachment and securing the position of the parasite among the secondary gill lamellae, somewhat analogous to a zip fastener [11]. Less prominent tegumentary folds and three highly mobile lobes have been described on the haptor of *P. homoion* [15]. The tegument of platyhelminths is reported as apically bound by a plasma membrane (generally described as a trilaminar), underlain by a thick nuclear-free syncytium layer packed with numerous mitochondria, secretory bodies and other inclusions, while the underside of the syncytium is lined with a basal plasma membrane. A fibrous basal lamina supports the entire structure. The basal plasma membrane produces numerous invaginations toward the syncytium, thereby forming a basal labyrinth [17]. Although we confirmed the general organisation of the tegument in *E. nipponicum* using standard TEM methods, freeze-etching revealed some differences in membrane organisation. The apical plasma membrane previously described as “trilaminar” actually corresponds to a plasma membrane closely associated with a subjacent membrane and separated by a dense layer of proteins. Similar observations were reported in a freeze-fracture study on the tegument of *Schistosoma mansoni* Sambon, 1907 [17]. Obviously, these two membranes are difficult to detect under conventional TEM. In addition, although a membrane-like structure was detected in ultrathin sections, no basal plasma membrane was detected in the proximity of freeze-fractured basal lamina (between the innermost site of the syncytium and the basal lamina); instead, the outer surface of the basal lamina was covered with a number of separating vesicles and projections of endoplasmic reticulum. Prominent body wall musculature separates the basal lamina from the cell bodies (the so-called subtegumentary cells, precursors or cytons) with prominent nuclei that are linked to the syncytium via numerous cytoplasmic projections and reach up to the body parenchyma [17, 19].

Smyth and Halton [17] considered the tegument of monogeneans to be a secretory epithelium, producing various vesicular and granular inclusions for dispersal on the surface. These authors also suggested that some synthetic activity occurs directly in the syncytium, due to the presence of abundant complexes of granular endoplasmic reticulum and Golgi apparatus in this area. In addition to the endoplasmic reticulum projections on the outer surface of the basal lamina, freeze-etching in this study revealed the presence of vesicles connected to the subjacent membrane, or even interrupting both superficial membranes covering the outer site of the syncytium (Fig. 6C-E). As the presence of accumulated vesicles or bodies within the syncytium has been observed in replicas and histological sections, we consider these to be secretory. Similar vesicles, considered to be exocytotic, were detected at the apical surface of the syncytium in ultrathin sections of *Pricea multae* Chauhan, 1945 [20]. In cestodes, pore-like openings in the protoplasmic face of the basal plasma membrane were considered to be pits from which the membrane-bound channels, extending into the syncytium and opening at its apical surface, emerged, and that these may facilitate transfer between the parenchyma and the parasite surface [21]. Accordingly, other studies identified large cells (considered glands) located within the parenchyma containing abundant electron-dense secretory vesicles and mitochondria, with an opening extending from the subtegumental musculature to the outer syncytial layer [22]. The contents of the vesicles are believed to release externally at the apical surface through these openings. Though the chemical composition and role of such secretory substances (which may differ among species) remains unclear, one theory attributes them to the secretion of a protective glycocalyx, as known for digeneans [17]. In this study, we observed no glycocalyx layer or coat covering the tegument surface; instead, we revealed a dense layer of proteins located between the apical plasma and subjacent membranes. It should be mentioned, however, that the glycocalyx layer could have been washed off during the numerous sample processing steps for electron microscopy and freeze-etching. In addition, this layer is often not visible until stained with ruthenium red or alcian blue (cationic reagents for electron microscopy) (e.g. Figure 5B and C vs. 5F in [23]). Interestingly, another study on diplozoids reported the existence of so-called scales, located mainly on the hindbody ridges just beneath the apical plasma membrane [14]. However, we believe that these “scales” correspond to areas of the trilaminar surface with more oblique sectioning, comprising two membranes (apical plasma and subjacent membrane) separated by a dense protein layer (as mentioned above) of various thickness. Our assumption is further supported by the fact that the trilaminar layer in the majority of shown TEM micrographs of higher magnification revealed areas of differing thickness [14]. These formations, corresponding to the local thickenings of the trilaminar surface and more prominent in the hindbody ridges, are also present in our micrographs (compare Figs. 3D-E vs. 7 J). Such formations may be due to increased accumulation of proteins in the middle layer and may be related to the (potentially) increased adhesion of this hindbody part.

In accordance with the study on *P. homoion* [15], hydrochloric carmine staining for CLSM and conventional histological staining of *E. nipponicum* revealed numerous giant cells with prominent nuclei between the GMO, below the buccal suckers and surrounding the pharynx. As these exhibited some differences from parenchymal cells, such as cytoplasm intensively staining with haematoxylin (stains basophilic structures) and netlike chromatin radially attached to the nucleolus (vs. more homogeneous chromatin distribution in parenchymal cells; shown in Fig. 2C), they are generally considered to be unicellular glands [6]. Further, they are at least two times larger than parenchymal cells and their nucleolus stains intensively pink with haematoxylin-eosin staining, in contrast to the purple nucleoli in parenchyma (personal observation). Similar gland cells, described in other monopisthocotyleans, have been shown to secrete a glutinous or sticky material most likely involved in adhesion to the host’s gills, though some of the secretory products may also modulate a host’s immune reaction [22, 24]. Alternatively, the gland cells could secrete pheromones [17]. GMO are also likely to be involved in secretion, though their presence has only been confirmed in developmental stages of *E. nipponicum* following fusion of diporpae [6]. These apparently hollow organs appear to open ventrally into the corner of the mouth cavity and possibly act as product reservoirs for neighbouring gland cells. Although their glandular function has not been confirmed thus far, the GMO are considered be a part of the digestive tract (e.g. [6, 25]). While we failed to observe any connection with other internal organs in a previous study [6], CLSM using transmission light mode in this study revealed a single long curved canal starting from the GMO and leading to the area above the buccal sucker (Fig. 2B). Previous work focused on the nerve system of *E. nipponicum* has suggested that the GMO are active structures as they are adequately innervated [9]. It has previously been speculated that the GMO act either during feeding or serve as the reservoir of secretions produced in gland cells found near the GMO [6]; however, the absence of GMO in non-fused diporpae exhibiting food intake is at odds with their involvement in parasite feeding. Two apical groups of symmetrically organised, F-actin rich circular structures of unknown function were repeatedly detected in *E. nipponicum* and *P. homoion* in the area of apical round projections observed under SEM [6, 15]. Accumulations of numerous drop-like gland cells located in the forebody apical area and between the GMO suggests that these circular structures could in fact be the openings of their drainage canals. However, their involvement in neurosensory activity cannot be ruled out as a pair of sensory nerves appear to interfere with these structures [5, 9]. While the central large circular structure could be the circular rim of a neurosensory receptor [15], the surrounding smaller circles more likely correspond to the outlet of joined glands, similar to those forming the anterior adhesive apparatus in other monogeneans [26–29]. Furthermore, smaller gland-like cells, located within the parenchyma and free secretory bodies (most likely secreted from a gland cell) in the syncytium, have been observed in the haptor region of *E. nipponicum* and *P. homoion* [14, 15], this study. We assume that secretory bodies in this area serve to transport adhesives from the gland cells to the surface of the haptor. Similar dense secretory bodies, produced by uninucleated gland cells and released to the surface via ducts, have been described in other species [22, 30, 31]. Further, monogeneans equipped with haptoral anchors [30, 31] display similar dense bodies in the syncytial multinucleated haptor gland (hamulus gland). These produce a liquid exudate that is stored in the hook reservoir until leaking into the sleeve cavity to bath the naked hook*.* The role of the hamulus gland is thought to be histolytic, facilitating penetration of gill tissue [31]. Other studies have proposed that a secretion exocytosed onto its surface gives the haptor adhesive properties [32].

A recent study on *E. nipponicum* combining laser capture microdissection with mass spectrometry [33] identified 2059 proteins (including 72 peptidases and 33 peptidase inhibitors) in the intestine (1978), tegument (1425) and parenchyma (1302). It is possible that the tegumental proteins may be located within the dense protein layer visualised in freeze-etching replicas and/or the above-mentioned tegument thickenings, while the parenchymatic proteins may accumulate within the giant, possibly secretory, cells and/or the secretory bodies.

The excretory (protonephridial) system of *E. nipponicum* is of typical organisation and similar to that in other platyhelminths. The role of the terminal cells in protonephridia (termed flame cells) is still not completely understood; however, they are likely to function in excretory/secretory processes and/or participate in maintenance of the osmotic environment [34]. In addition to conventional TEM (described elsewhere [12, 35]) and CLSM-based tubulin labelling [15], this study is the first to use the freeze-etching technique to visualise the flame cells, their tufts and the protonephridial duct in monogeneans. Aside from the remarkably prolonged lamellae lining the protonephridial and the excretory ducts, the ultrastructure of the protonephridial system generally corresponds to that already published for diplozoids [12]. These lamellae have previously been reported as an extensive reticulum formed by numerous interconnected cavities within the epithelium of the protonephridial duct [36]. According to other works on platyhelminths [36, 37], lateral flames were observed within the protonephridial ducts. The organisation of the flame bulb corresponds to that observed in other monogeneans (except *Udonella*), aspidogastreans and digeneans [36, 38].

### Niche searching and sensing the host environment

In addition to the posteriorly located haptor, most monogeneans are thought to use their anterior ends (for diplozoids, specifically the buccal suckers) for transient attachment to the host (e.g. during feeding or translocation on the host’s gills) [6]. It is likely that all monogenean ectoparasites can change their location on the host via leech-like locomotion based on temporary attachment of the apical forebody during detachment and relocation of the haptor [32]. In monogeneans studied thus far (e.g. *Leptocotyle*), this leech-like locomotion is usually achieved through cooperation between adhesive secretion (discussed above) and the haptor [39].

In *E. nipponicum*, the order of individual muscle layers appears to differ slightly from the generalised muscle organisation (i.e. outer circular, intermediate diagonal and inner longitudinal muscle fibres) of parasitic worms [40]. In *E. nipponicum*, we documented outer circular and inner longitudinal muscles interwoven by diagonal muscle bundles in a basket-like manner. In *P. homoion*, similarly perpendicular muscles were anchored to the tegument [15]. These appear to correspond to the so-called dorsoventral muscles [41], whose function may be related to the dorsoventral flattening of the worm. Also, of interest were the abundant muscle fibres located within the tegumentary ridges, which could make it easier for the worm to attach itself between the secondary gill lamellae.

While a basic diagram showing the nervous system of *E. nipponicum* was published as early as 1891 [25], later immunomicroscopic studies focusing on the central nerve elements in paired adults demonstrated peptidergic and serotoninergic innervation (via indirect immunocytochemistry), cholinergic components (via enzyme cytochemistry) and neuropeptide immunoreactivity at the subcellular level (via TEM immunogold) [9]. In this study, in addition to identifying components of the central nervous system, we were able to visualise the network of peripheral nerves and innervation of sensory structures using α-tubulin labelling for CLSM. These results are generally consistent with our observations on *P. homoion* [15]. Furthermore, this study was also able to provide visualisation of the peripheral nerve fibres, along with the innervation of sensory structures and clamps, using TEM and freeze-etching TEM. The fine structure of the nerve fibres corresponds with that observed in other studies on monogeneans [14, 20, 42, 43] and other platyhelminths [44, 45].

Diplozoid sensory structures, which appear to be numerous and of several types, may serve for the evaluation of external and internal conditions and/or assist in the search for a competent host. In accordance with our study on *P. homoion*, no microvilli (reported on the surface of some monogeneans) are present on the body surface of *E. nipponicum* (except for the buccal cavity) and we confirmed that the well-innervated sensory structures are distributed over the entire body, despite being concentrated in the forebody and hindbody [11, 15]. The single uniciliated receptors with a tegumentary rim accumulated around the mouth opening, while also occurring on the *E. nipponicum* and *P. homoion* forebody, appear to be involved in contacting the host tissue and subsequent feeding [11, 15]. These structures are generally believed to have a tango- and/or rheoreceptory function [17]; however, in skin monogeneans, it has been suggested that they may function as mechanoreceptors detecting turbulence or vibration in water currents generated by fish in close proximity [46]. Generally speaking, the uniciliated receptor architecture corresponds with that in other monogeneans [13, 15, 20, 46–48]. Of particular interest is the organisation of the base in receptors with a prominent tegumentary rim (Fig. 9I), which differs in having much more massive innervation and the presence of muscles. Similar raised receptors with muscles surrounding the central innervation were detected by immunofluorescence (inset in Fig. 10B). It seems likely that such musculature would allow receptor positioning. In SEM micrographs of *P. homoion*, the cilium of single uniciliated receptors was seen to be anchored by radially organised septa embedded in the tegument [15]. In this study, similar radial septa were occasionally detected in ultrathin sections of *E. nipponicum*. These appear to correspond to the transitional striated fibres arising from the basal body of the cilium (in a similar manner to wheel spokes) and extending to the dense collar of the nerve bulb [49]. In addition to uniciliated sensory structures, both this study and previous studies have shown that the lateral side of the hindbody in *E. nipponicum* bears a row of non-ciliated papillae that we assume are involved in reception of environmental stimuli [11, 15]. Similar non-ciliated receptors, including the clusters of papillae above the clamps in *P. homoion* [15] and papillae in *Entobdella soleae* (Van Beneden & Hesse, 1863) [2, 48], are generally assumed to function as mechanoreceptors while contacting the host [17]. In our previous study, we suggested that the proprioreceptors may have an alternative role in sensing the relative position of the haptor during movement [15].

## Conclusions

Here, we provide a comprehensive microscopic analysis of the tegument and related structures of *E. nipponicum*. Freeze-etching enabled us to detect some differences when compared to observations made using conventional TEM, including the lack of a basal plasma membrane and the presence of a membrane subjacent to the apical plasma membrane and separated by a dense protein layer. Hydrochloric carmine staining revealed the locations of unicellular glands accumulated predominantly in the apical forebody and hindbody haptor region. Vesicles and dense bodies were localised and discussed in relation to secretory/excretory processes. The order of individual muscle layers in *E. nipponicum* differed slightly from the organisation of body wall musculature generalised for parasitic worms in that the outer circular and inner longitudinal muscles were interwoven with diagonal muscle bundles, with additional perpendicular muscles anchored to the tegument. Of particular interest were the abundant muscles filling the tegumentary ridges, which most likely assist in fixing the parasite between the gill lamellae. By using freeze-etching alongside conventional TEM and CLSM-based tubulin labelling, we were able to visualise the protonephridial system and provide better visualisation of some components of the central nervous system, the network of peripheral nerves and innervation of receptors. We identified three types of receptors: uniciliated sensory endings with a subtle (or missing) tegumentary rim; obviously raised uniciliated receptors with a prominent tegumentary rim (packed with massive innervation and muscles) and non-ciliated papillae (restricted to the hindbody lateral region). Our study of *E. nipponicum* points to specific morphofunctional adaptations that have evolved in ectoparasitic monogeneans for confrontation of host fish. A deeper understanding of the parasitism strategies/adaptations of monogeneans and their specific behavioural features (e.g. their host-searching activity, invasive and pathogenic mechanisms, virulence factors along with corresponding structures and parasite’s self-protection mechanisms) could help explore practical applications such as controlling (or even eradicating) the serious pathogens from commercial fish cultures. In addition, diplozoid monogeneans represent a model that is indeed suitable for various studies on host-parasite systems, the findings of which could be applied in other flatworm groups of economic or medical importance.

## Methods

### Material collection

Samples of *E. nipponicum* were collected from the gills of naturally infected common carp (*C. carpio*) caught by electrofishing or gillnets in the littoral zone of the Mušov lowland reservoir (Czech Republic; 48° 53′ 12″ N, 16° 34′ 37″ E). Fish collection was carried out by external collaborators from Institute of Vertebrate Biology of the Czech Academy of Sciences, Czech Republic (wild fish collection by the Institute of Vertebrate Biology is approved via Ministry of Agriculture certificate No. 3OZ31162/2011–17,214). The fish were transported in aerated original water to the laboratory facilities of the Faculty of Science at Masaryk University, Brno, Czech Republic (Permit No. 16256/2015-MZE-17214). Fish (*n* = 38) were sacrificed by first stunning (a forceful and accurate blow to the head with a blunt instrument) and then cutting the spine and the main artery, with all efforts made to minimise suffering (in accordance with Act No. 246/1992 Coll., on the Prevention of Cruelty to Animals). The gills were removed according to standard protocols [50] and checked for ectoparasites using fine needles under an Olympus SZX7 zoom stereo microscope. Living parasites were taxonomically identified [51] using an Olympus BX51 light microscope.

### Histology

Samples of *E. nipponicum* (25 adult worms) were fixed in alcohol-formalin-acetic acid (AFA fixative) and processed using standard histological methods [6]. Sections 5–7 μm thick were stained with green Masson’s trichrome (MT) [52] and examined under an Olympus BX51 microscope.

### Confocal laser scanning microscopy

Samples of *E. nipponicum* (14 adult worms) were flat-fixed between microscopic slides in a freshly prepared 4% paraformaldehyde in 0.1 phosphate buffered saline (PBS) for 4 h at 4 °C and then transferred into fresh fixative. For direct labelling of filamentous actin, specimens were washed for 24 h in antibody diluent (AbD) containing 0.1 M PBS, 0.1% bovine serum albumin, 0.5% Triton X-100 and 0.1% NaN_3_ (pH 7.4). The samples were then incubated in phalloidin-tetramethylrhodamine B isothiocyanate (phalloidin-TRITC; Sigma-Aldrich, Czech Republic) in AbD (10 μl/1 ml) for 48 h at 4 °C, then washed again in AbD for 24 h at 4 °C. For indirect antibody labelling, specimens were permeabilised for 48 h in 0.5% Triton X-100 (Sigma-Aldrich), incubated for 4 days at 4 °C in mouse monoclonal anti-α-tubulin antibody (Sigma-Aldrich, Czech Republic), washed for 24 h in AbD and incubated with mouse polyvalent immunoglobulins (1:125) in PBS with 1% BSA at 37 °C for 3 days. Some specimens were subsequently washed and incubated in TRITC-phalloidin. Controls were incubated with FITC-conjugated secondary antibody without the primary antibody. Some preparations were counterstained with Hoechst for localisation of cell nuclei. For hydrochloric carmine staining, whole-mount preparations of worms were fixed in 70% ethanol, stained and differentiated in acidic 70% ethanol [53]. All preparations were finally mounted in VECTASHIELD® (Vector Laboratories, USA). The samples were examined under an Olympus IX81 microscope equipped with a laser scanning FluoView 500 confocal unit (Olympus FluoView 4.3 software). Fluorescent labelling was visualised using the 405 Diode (405 nm; Hoechst), Argon (488 nm excitation; anti-α-tubulin-FITC) and He/Ne green (543 nm excitation; phalloidin-TRITC and hydrochloric carmine) lasers.

### Electron microscopy

For transmission electron microscopy (TEM), the specimens (56 adult worms) were fixed in 3% (v/v) glutaraldehyde in 0.1 M cacodylate buffer (pH 7.3) for 2 h, washed in 0.2 M cacodylate buffer and post-fixed in 1% (w/v) OsO_4_ in the same buffer for 2 h at 4 °C. The specimens were then dehydrated in a graded ethanol series and propylenoxide before embedding in Epon/Araldite (Araldite® 502/PolyBed® 812 Kit, Polysciences). Sections were cut with glass knives and stained with uranyl acetate and lead citrate. Observations were made using the Morgagni 268 D (FEI) TEM.

For scanning electron microscopy (SEM), specimens (14 adult worms) were carefully washed in freshwater to remove fish mucus and fixed in either hot 4% formaldehyde or 4% glutaraldehyde at 4 °C for 24 h and processed according to published protocols [11]. The preparations were examined in a JEOL 6300 or VEGA TESCAN SEM operating at 15 kV.

### Freeze-etching

Parasites (10 adult worms) were fixed at 4 °C in 3% (v/v) glutaraldehyde in 0.1 M cacodylate buffer (pH 7.3), washed in the same buffer and processed according to published protocols [54] using a BAF 060 freeze-etching system (BAL-TEC). Replicas were cleaned with 5% sodium hypochlorite, 90% sulphuric acid and 90–95% chromo-sulphuric acid, washed in distilled water and mounted on copper grids for examination using a Morgagni 268 D TEM (FEI).

## Supplementary Information

**Additional file 1. **Tegument organisation in diporpa and juvenile stage of *Eudiplozoon nipponicum*. **A.** General view of a diporpa tegument. TEM. **B.** General view of a juvenile tegument. TEM*. apm* – apical plasma membrane, *bl* – basal lamina, *bpm* – basal plasma membrane, *m* – body wall musculature, *n* – nucleus, *p* – parenchyma, *sc* – syncytium.

## Data Availability

All data generated or analysed during this study are included in this published article and its additional file. The raw datasets are available from AV and IH on reasonable request.

## Abbreviations

AbD: Antibody diluent
CLSM: Confocal laser scanning microscopy
FE: Freeze-etching
FITC: Fluorescein isothiocyanate
GMO: Glandulo-muscular organs
LM: Light microscopy
MT: Masson’s trichrome
PBS: Phosphate buffered saline
SEM: Scanning electron microscopy
TEM: Transmission electron microscopy
TRITC: Tetramethylrhodamine isothiocyanate
