# Supplementary figures and images for: Eudiplozoon nipponicum: morphofunctional adaptations of diplozoid monogeneans for confronting their host
Source: BMC Zool. 2021 Aug 17;6:23. doi: 10.1186/s40850-021-00087-5 (PMC10127055; doi:10.1186/s40850-021-00087-5)

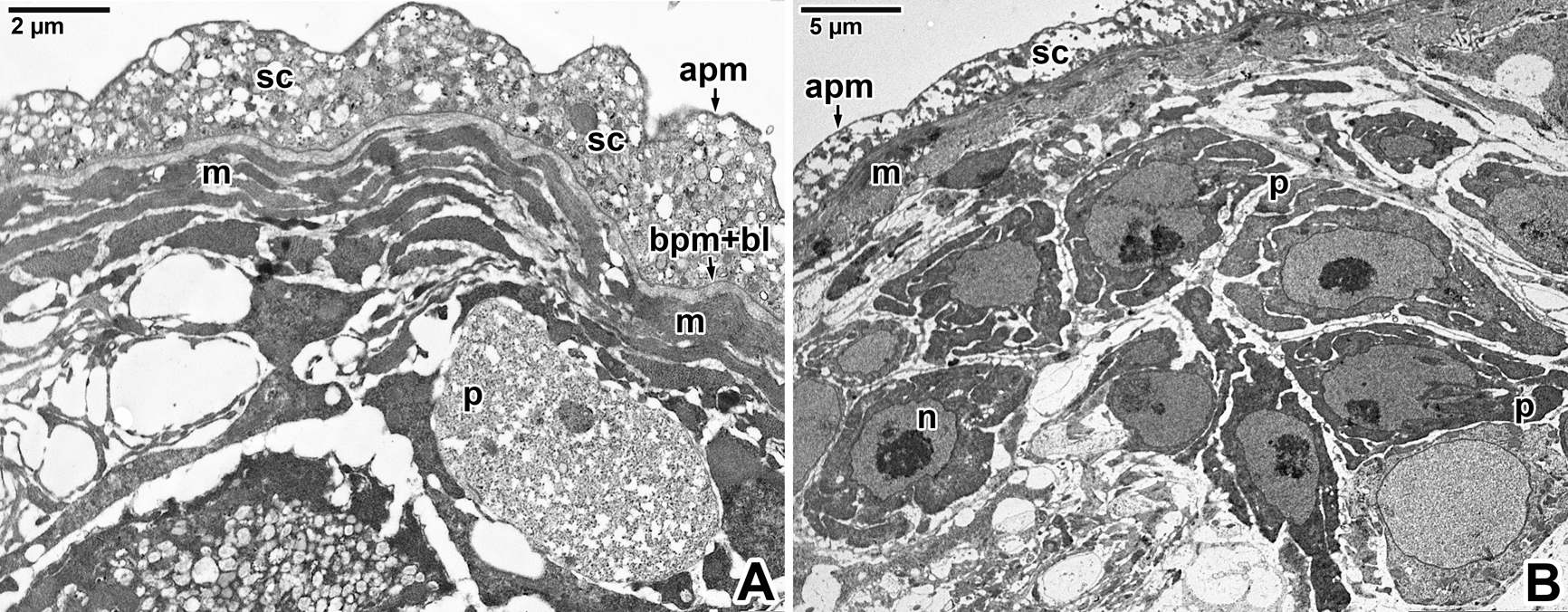

Supplement: Supplementary file 1 — Additional file 1. Tegument organisation in diporpa and juvenile stage of Eudiplozoon nipponicum. A. General view of a diporpa tegument. TEM. B. General view of a juvenile tegument. TEM. apm – apical plasma membrane, bl – basal lamina, bpm – basal plasma membrane, m – body wall musculature, n – nucleus, p – parenchyma, sc – syncytium. [file 40850_2021_87_MOESM1_ESM.tif]
